# Complete Freund’s Adjuvant Induces a Fibroblast-like Synoviocytes (FLS) Metabolic and Migratory Phenotype in Resident Fibroblasts of the Inoculated Footpad at the Earliest Stage of Adjuvant-Induced Arthritis

**DOI:** 10.3390/cells12060842

**Published:** 2023-03-08

**Authors:** Susana Aideé González-Chávez, Eduardo Chaparro-Barrera, María Fernanda Alvarado-Jáquez, Rubén Cuevas-Martínez, Rosa Elena Ochoa-Albíztegui, César Pacheco-Tena

**Affiliations:** 1 Laboratorio PABIOM, Facultad de Medicina y Ciencias Biomédicas, Universidad Autónoma de Chihuahua, Chihuahua 31125, Mexico; 2 Radiology Department, Memorial Sloan Kettering Cancer Center, 1275 York Ave, New York, NY 10065, USA

**Keywords:** fibroblast-like synoviocytes, fibroblast migration, cadherin-11, type VI collagen

## Abstract

The fibroblast-like synoviocytes (FLS) have a crucial role in the pathogenesis of Rheumatoid Arthritis (RA); however, its precise mechanisms remain partially unknown. The involvement of the fibroblast in activating adjuvant-induced arthritis (AA) has not been previously reported. The objective was to describe the participation of footpads’ fibroblasts in the critical initial process that drives the AA onset. Wistar rats were injected with Complete Freund’s Adjuvant (CFA) or saline solution in the hind paws’ footpads and euthanized at 24 or 48 h for genetic and histological analyses. Microarrays revealed the differentially expressed genes between the groups. The CFA dysregulated RA-linked biological processes at both times. Genes of MAPK, Jak-STAT, HIF, PI3K-Akt, TLR, TNF, and NF-κB signaling pathways were altered 24 h before the arrival of immune cells (CD4, CD8, and CD68). Key markers TNF-α, IL-1β, IL-6, NFκB, MEK-1, JAK3, Enolase, and VEGF were immunodetected in fibroblast in CFA-injected footpads at 24 h but not in the control group. Moreover, fibroblasts in the CFA inoculation site overexpressed cadherin-11, which is linked to the migration and invasion ability of RA-FLS. Our study shows that CFA induced a pathological phenotype in the fibroblast of the inoculation site at very early AA stages from 24 h, suggesting a prominent role in arthritis activation processes.

## 1. Introduction

Fibroblast-like synoviocytes (FLS) are relevant players in the pathogenic process of Rheumatoid Arthritis (RA) [1,2,3]; they mediate the altered dynamics of the rheumatoid synovium and perpetuate RA. RA-FLS phenotype requires the transformation of an otherwise quiescent structural cell into an aggressive and proliferative cell, which interacts with- and activates immune cells [4,5,6,7,8]. Currently, FLS is considered a potential therapeutic target for RA patients [9,10,11].

RA-FLS exhibit a metabolic profile parallel to some tumors, thriving in a hypoxic environment with increased production of oxygen radicals and cytokines [12,13,14]. Under stressful conditions, FLS has the potential to initiate, coordinate, and perpetuate inflammatory processes through complex interactions with adaptive immune response cells [5,15,16,17,18].

Several surface markers of FLS and its pathogenic signaling pathways have been characterized [19,20,21]. Nevertheless, the origin of RA-FLS and the mechanisms driving the transformation of their precursors remain largely undefined. Very likely, FLS derive either from mesenchymal stem cells (MSC) or synovial resident fibroblasts. Furthermore, the fact that FLS and other mesenchymal-derived cells can migrate and disseminate in the bloodstream raises the possibility that either FLS or activated precursors can be induced elsewhere and settle on joints, expanding the inflammatory process [22,23,24].

Adjuvant-induced arthritis (AA) rat model resembles human RA. Complete Freund’s Adjuvant (CFA) injections in the footpads are enough to induce joint swelling, synovial lymphoid infiltrate, and joint destruction [25,26]. AA is a standard testing field for novel treatments for RA [27,28,29,30], confirming the similarity of their pathogenesis. However, despite understanding the downstream consequences of the CFA injection, the initial mechanisms to induce arthritis onset remain unknown, especially at the injection site at the earliest events. Since it is a controlled scenario, AA can allow the detection of essential mediators at the earliest stages that lead to arthritis and which are probably undetectable once arthritis sets on.

Therefore, the present study aimed to describe the metabolic and immune profile triggered by the CFA in the subcutaneous injection site, within the resident cells, including fibroblast in the AA model’s earliest stages. The transcriptome modifications were evaluated by microarray technology and its subsequent bioinformatic analysis. Additionally, the role of local fibroblasts in the injected site was confirmed by the immunodetection of metabolic and immune markers.

## 2. Materials and Methods

### 2.1. Animal Model

The study included 24 8-week-old male Wistar rats that were initially randomly divided into 2 groups of 12 rats each: the CFA group that received the injection of 0.3 mL of CFA (1 mg *Mycobacterium tuberculosis* (H 37RA, ATCC 25177), heat-killed and dried, 0.85 mL paraffin oil, and 0.15 mL mannide monooleate; Sigma Aldrich Chemic, cat. No. F5881) in the hind paws’ footpads [31]; and the control group that received the same volume of saline solution (SS). Rats were kept under controlled luminosity conditions (12 h light/12 h dark) and temperature (23 ± 2 °C) and received food and water *ad libitum*. Six rats from each group were euthanized with isoflurane 24 h after the CFA or SS injection, and the other 6 from each group 48 h after the injections. Finally, 4 study groups were formed: CFA-24 h, CFA-48 h, SS-24 h, and SS-48 h. Footpads were dissected for RNA extraction and histological analysis (1 of the hind paws of each rat for each experimental strategy). The entire protocol, including manipulation of the animals, complied with the institutional ethics committee and Institutional Animal Care and Use Committee (IACUC), ID numbers: UACH, CEI-B/329/15, UACH, FM-FM-EXT-B-339/17.

### 2.2. DNA Microarray and Bioinformatics Analysis

After the euthanasia, injected footpads (6 per study group) were pulverized on liquid nitrogen using a mortar and pestle. Total RNA was purified using the RNeasy^®^ Lipid Tissue Mini Kit (Qiagen, Hilden, Germany) extraction kit, following the manufacturer’s protocol. RNA concentration and integrity were verified on a Bioanalyzer 2100 (Agilent Technologies, Santa Clara, CA, USA). For the microarray assay, the RNA of each study group was mixed in equimolar quantities to form the pools used in the DNA microarrays.

The DNA microarrays were performed Institute of Cellular Physiology, Autonomous University of Mexico (UNAM), Mexico. The expression profiles of CFA (experiment) vs. SS (reference) at 24 and 48 h were evaluated. The microarrays included the reverse transcription-polymerase chain reaction (RT-PCR), and the resulting cDNAs were labeled with Cy5 (CFA group) or Cy3 (SS groups). Hybridization was done in the Rn5K (UNAM, Mexico) chip containing 5000 rat genes. The signal was scanned and acquired using the ScanArray 4000 (Packard BioChips Technologies, Billerica, MA, USA) and analyzed in the GenArise Microarray Analysis Tool software (UNAM, Mexico). The lists of differentially expressed genes (DEGs) [Z-score ≥ 1.5 standard deviations (SD)] in the CIA-injected respect SS-injected footpads were obtained.

The list of DEGs was further analyzed in DAVID Bioinformatics Resources 6.8 (https://david.ncifcrf.gov/ accessed on 2 March 2023), an open-resource platform that classifies genes list into functional biological processes and KEGG (Kyoto Encyclopedia of Genes and Genomes) signaling pathways [32]. Furthermore, DEGs were also analyzed on the STRING 11.5 database (https://string-db.org/ accessed on 2 March 2023) to obtain the analysis and integration of direct and indirect protein-protein interactions (IPP) centered on the functional association [33]. The DEGs identified in the microarray were loaded, and the interactions with minimal confidence (interaction score > 0.4) were selected. Finally, the obtained IPP network was analyzed more thoroughly to obtain primary clusters of sub-networks using the Cytoscape software (version 3.9.1) with the Molecular Complex Detection (MCODE) complement (node score cutoff = 0.4) [34,35]. The main clusters were analyzed in STRING to obtain the IPP networks and their associated KEGG signaling pathways. Those pathways and genes of relevance in arthritis were identified.

### 2.3. Histological Analysis

The injected hind paws (six per study group) were fixed in 10% formaldehyde, decalcified with 5% nitric acid for 24 h, dehydrated in ethanol, and embedded in paraffin [36]. Sections of 3 μm were obtained and stained with hematoxylin and eosin (H&E) for histological evaluation. The images were acquired with a digital camera coupled to the optical microscope (AxioStar plus, Carl Zeiss, Berlin, Germany). The histological analysis was performed in the subcutaneous injection site and not the synovial structures because we wanted to explore the effect of CFA within the injected tissues.

Immunohistochemistry was done with specific antibodies against rat cell surface receptors as well as intracellular signaling molecules such as CD4 (11-0042-82), CD8 (14-0081-82), CD68 (14-0689-82; eBioscience^TM^, Invitrogen, Waltham, MA, USA), tumor necrosis factor (TNF)-α (sc-52746), Interleukin (IL)-1β (sc-32294), IL-6 (sc-130326), nuclear factor kappa-light-chain-enhancer of activated B cells (NF-κB; sc-8414), Toll-like receptor (TLR)-4 (sc-10741), mitogen-activated protein kinase kinase (MEK)-1 (sc-6250), Janus kinase (Jak)-3 (sc-513), enolase (Eno; sc-100812), and vascular endothelial growth factor (VEGF; sc-7269; Santa Cruz Biotechnology, Dallas, TX, USA). Tissue sections were deparaffinized in xylene and dehydrated in descending ethanol until water. Antigen retrieval was done using 0.05% trypsin (T1426-250 mg, SIGMA Life Science, St. Louis, MO, USA) for 30 min at 37 °C or 1 mM EDTA pH 8.0 for 30 min at 95 °C. The slides were treated with 0.2% Triton-X100 (Bio-Rad, Hercules, CA, USA). After blocking with 10% bovine serum albumin (BSA; A9647-100G SIGMA Life Science, St. Louis, MO, USA) for 1 h at room temperature in a humidified chamber, the tissues were incubated with the primary antibody in a 1:200 dilution at 4 °C overnight. The corresponding isotype’s biotin-streptavidin-conjugated secondary antibodies (Jackson ImmunoResearch Laboratories, Inc., West Grove, PA, USA) were used in a 1:400 dilution. Immunodetection was carried out using the Pierce^®^ streptavidin horseradish peroxidase-conjugated (Jackson ImmunoResearch Laboratories, Inc., West Grove, PA, USA) and Diaminobenzidine (DAB; D4293-50SET, SIGMA-ALDRICH, USA) as the chromogen. The primary antibody was replaced with PBS buffer to establish negative controls. Images were acquired using a digital camera (AmScope MU1803, Irvine, CA, USA) and an optical microscope (AxioStar Plus, Carl Zeiss, Berlin, Germany). The expression of CD4, CD8, and CD68 at 24 h and 48 h was quantified with the ImageJ program and the IHC toolbox. The DAB color was extracted from each image, and the maximum and mean gray values were obtained. Each image’s optical density (OD) was obtained with log10 (maximum gray value/mean gray value). The OD’s means and SD were calculated and graphed per study group.

The double indirect-immunofluorescence (IIF) was performed to co-localize the TNFα, IL-1β, IL-6, NFκB, TLR-4, MEK-1, JAK3, Eno, VEGF, and cadherin-11 (CDH11) markers in the fibroblasts of the CFA-injected hind paws. The immunofluorescence was performed sequentially. First, the tissues were deparaffinized, and antigen retrieval was performed with 0.05% trypsin for 30 min at 37 °C or 1 mM EDTA pH 8.0 for 30 min at 95 °C. Subsequently, the tissues were permeabilized with 0.2% Triton-X100 and blocked with 5% normal donkey serum. Next, sections were incubated with the first primary antibody (1:200 dilution): CD4 (11-0042-82), CD8 (14-0081-82), CD68 (14-0689-82; eBioscience^TM^, Invitrogen, USA), TNFα (sc-52746), IL-1β (sc-32294), IL-6 (sc-130326), NF-κB (sc-8414), TLR-4 (sc-10741), MEK-1 (sc-6250) Jak-3 (sc-513), Eno (sc-100812), VEGF (sc-7269), and CDH11 (sc-365867)(Santa Cruz Biotechnology, USA). After washing in PBS, tissues were incubated with the AF488-labeled secondary antibody (1:200 dilution): Donkey Anti-Mouse IgG (715-545-150) or Donkey Anti-Rabbit IgG (711-545-152; Jackson ImmunoResearch, USA). A second blocking was performed, and then tissues were incubated with the second primary antibody (1:200 dilution): Fibroblast Marker, ER-TR7 [against Collagen type VI (ColVI) [37,38] (sc-73355, Santa Cruz Biotechnology), washed in PBS, and incubated with the Cy5-labeled secondary antibody (1:200 dilution): Donkey Anti-Rat IgG (712-175-153, Jackson ImmunoResearch, USA). Labeling was evaluated by epifluorescence microscopy (Zeiss Axio Imager A1), and images were acquired using a digital camera (AmScope MU1203-FL, USA).

### 2.4. Statistical Analysis

The bioinformatics analysis of the microarray data included their statistical analysis. In DAVID, Fisher’s exact test measures gene enrichment in annotation terms. Fisher’s Exact *p*-values are computed by summing probabilities *p* over defined sets of tables (Prob = ∑Ap) [32]. In the STRING database, the PPI enrichment *p*-value indicates that the nodes are not random and that the observed number of edges is significant; for the associated-KEGG pathways, the false discovery rate (FDR) is defined as FDR = E(V/R|R > 0) P(R > 0) [39]. In Cytoscape-MCODE, the complex score is defined as the product of the complex subgraph, C = (V, E), density, and the number of vertices in the complex subgraph (DC × |V|) [35].

For OD measures in IHC, statistical analysis was made in SPSS statistics v22 software (SPSS Science Inc., Chicago, IL, USA). The Shapiro-Wilk and Kolmogorov-Smirnov tests were used to determine the data normality. In addition, means and SD were estimated and graphed, and a Student’s t-test was used to compare the expression of CD4, CD8, and CD68 at 24 h and 48 h. Differences were considered significant when *p* ≤ 0.05.

## 3. Results

### 3.1. DNA Microarray and Bioinformatic Analysis

**Figure 1:**
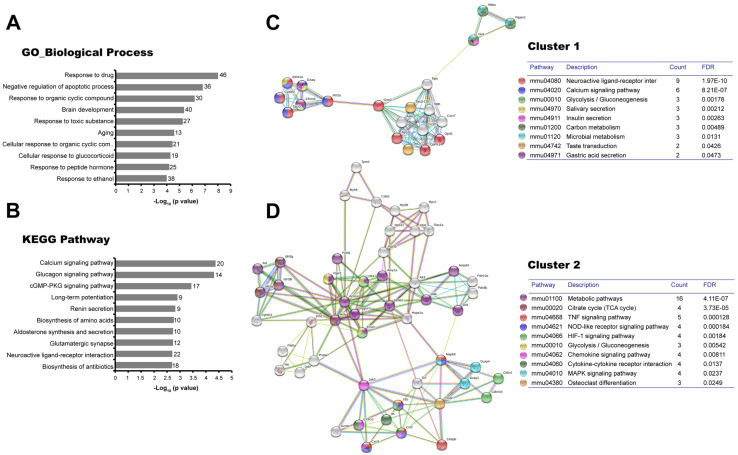
Transcriptomic dysregulation in hind paw footpads at 24 h post-Complete Freund’s Adjuvant injection. The lists of the differentially expressed genes (Z-score ≥ 1.5 SD) by CFA at 24 h were analyzed on the DAVID Bioinformatics Resources 6.8 (https://david.ncifcrf.gov/ accessed on 10 January 2023; (**A**,**B**)), STRING 11.5 database (https://string-db.org/ accessed on 10 January 2023) and Cytoscape 3.9.1 platforms (**C**,**D**). The top 10 Gene Ontology (GO) Biological Process (**A**) and the Kyoto Encyclopedia of Genes and Genomes (KEGG) pathways (**B**) resulting from the DAVID database are shown in bar charts indicating the −Log10 (*p*-value) of each GO and KEGG term. The number of genes involved in each term is shown on the right side of each bar. From the protein-protein interactions network of deregulated genes, the first (**C**) and second (**D**) clusters of sub-networks were obtained using the Molecular Complex Detection (MCODE) complement (cutoff = 0.4). The KEGG signaling pathways relevant to arthritis were selected (Tables) and marked with different colors. Line color indicates the type of interaction evidence. FDR: False discovery rate.

**Figure 2:**
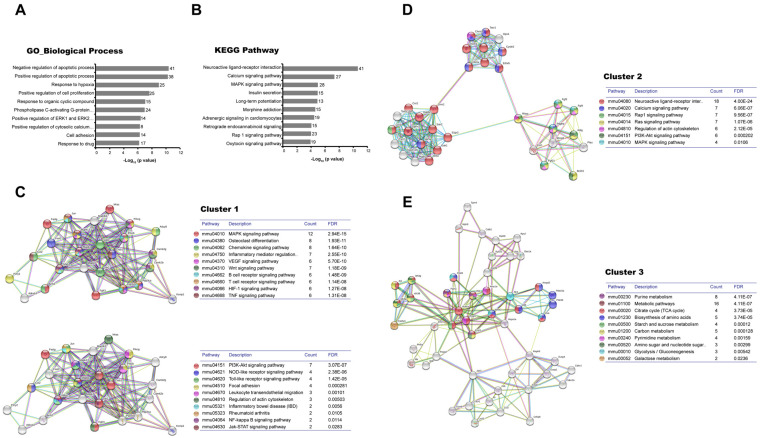
Transcriptomic dysregulation in hind paw footpads at 48 h post-Complete Freund’s Adjuvant injection. The lists of the differentially expressed genes (Z-score ≥ 1.5 SD) by CFA at 48 h were analyzed on the DAVID Bioinformatics Resources 6.8 (https://david.ncifcrf.gov/ accessed on 10 January 2023; (**A**,**B**)), STRING 11.5 database (https://string-db.org/ accessed on 10 January 2023) and Cytoscape 3.9.1 platforms (**C**,**D**). The top 10 Gene Ontology (GO) Biological Process (**A**) and the Kyoto Encyclopedia of Genes and Genomes (KEGG) pathways (**B**) resulting from the DAVID database are shown in bar charts indicating the −Log10 (*p*-value) of each GO and KEGG term. The number of genes involved in each term is shown on the right side of each bar. From the protein-protein interactions network of deregulated genes, the first (**C**), second (**D**), and third (**E**) clusters of sub-networks were obtained using the Molecular Complex Detection (MCODE) complement (cutoff = 0.4). The KEGG signaling pathways relevant to arthritis were selected (Tables) and marked with different colors. Line color indicates the type of interaction evidence. FDR: False discovery rate.

The microarray resulted in 663 DEGs (162 up/501 down) at 24 h after CFA injection and 689 DEGs (413 up/276 down) at 48 h. Furthermore, the bioinformatic analysis in the DAVID platform showed that these genes were significantly associated with biological processes (Figure 1A and Figure 2A) and KEGG pathways (Figure 1B and Figure 2B) related to RA, such as the response to hypoxia. The apoptosis regulation processes were associated with the highest number of genes at both times.

In the combined analysis with Cytoscape-MCODE at 24 h, the comparison between CFA and SS resulted in two relevant clusters. The first, which included 20 nodes, 75 edges, and a score of 7.89 (Figure 1C), mainly showed activation of intracellular signaling pathways, including neuroactive ligand-receptor interaction and calcium signaling pathways. Carbohydrate metabolism signaling pathways, including glycolysis, insulin secretion, and carbon metabolism, were also found in this cluster. The most relevant genes in this cluster were cholinergic receptor muscarinic 3 (Chrm3) and glutamate metabotropic receptor 2 (Grm2). The second cluster included 50 nodes, 163 edges, and a score of 6.65 (Figure 1D), which confirmed the relevance of metabolic pathways and showed several immune-relevant pathways mainly related to innate immunity, including TNF, NOD-like receptor, Chemokine, and Cytokine-cytokine receptor interaction signaling pathways. MAPK signaling pathway was also found dysregulated. The most prominent proteins in the network included Il1b, Mapk9, and several chemokines. The Jak3 and Eno3 were also relevant proteins in the network.

At 48 h, cluster 1, which included 25 nodes, 132 edges, and a score of 11.00 (Figure 2C), showed that both MAPK and PI3K-Akt signaling pathways were active. In addition, other pathways linked to human RA were present in the network, including HIF-1, TNF, VEGF, TLR, NOD-like receptor, Jak-STAT, and Wnt signaling pathways. The most interactive proteins in the network included several Mapk (1,13,7), Nfkb1, Jun, protein phosphatase three catalytic (Ppp3c) subunit alpha and beta, and protein kinase c (Prkc) beta and gamma. The second cluster included 33 nodes, 166 edges, and a score of 10.37 (Figure 2D), and the most significant signaling pathway was the neuroactive ligand-receptor interaction with 18 associated genes. This network also highlights the multiple interactions of fibroblast growth factors (Fgf) 3, 4, and 21 with the Rap1, Ras, MAPK, and PI3K-Akt signaling pathways.

**Figure 3:**
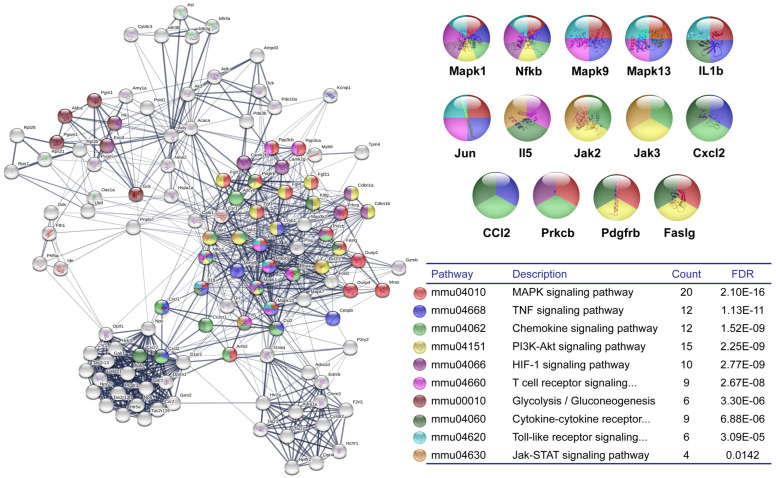
Rheumatoid Arthritis-related signaling pathways at the very early stages of adjuvant-induced arthritis. The genes of clusters 1 and 2 of the times 24 h and 48 h resulting from Cytoscape-MCODE analysis for each comparison were uploaded to the STRING platform to construct the protein interaction network. The KEGG signaling pathways relevant to arthritis were selected (Table) and marked with different colors. Nodes with the most significant interaction in the pathways of interest are amplified. FDR: False discovery rate.

The genes of clusters 1 and 2, both at 24 h and 48 h, were selected and analyzed in the STRING platform to construct the IPP network and highlight the related KEGG pathways at the earliest stages of the AA model. Figure 3 shows several pathways linked to RA, including HIF-1, TNF, MAPK, Chemokine, T-cell receptor, Jak-STAT, and Glycolysis/Gluconeogenesis, were associated with these genes. In addition, Mapk proteins (1 and 13) linked several protein circuits, and the Il1b was also a link between MAPK, TNF, TLR, and Cytokine-cytokine receptor interaction signaling pathways.

### 3.2. Histological Analysis

**Figure 4:**
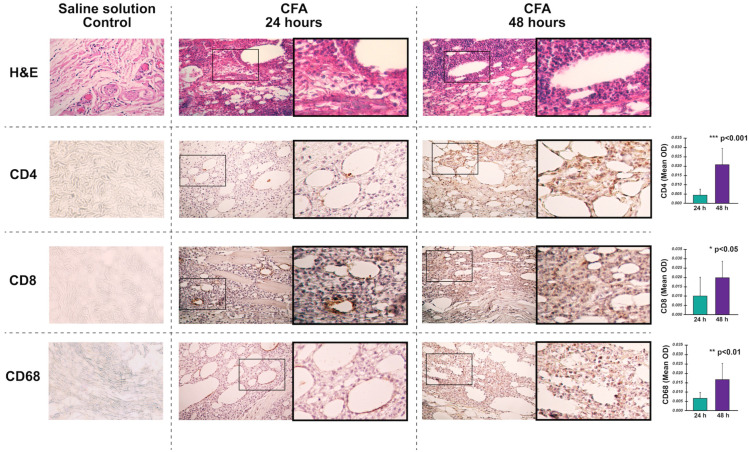
Histological analysis of the rats’ footpads in very early stages of adjuvant-induced arthritis. Inflammatory infiltrates in footpads after injecting a saline solution and Complete Freund’s Adjuvant (CFA) at 24 and 48 h through hematoxylin and eosin (H&E) stains are shown in Row 1. Immunodetection of CD4, CD8, and CD68 in CFA-injected footpads at 24 and 48 h are shown in Rows 2, 3, and 4, respectively. CD4, CD8, and CD68 expressions were quantified with the ImageJ program and the IHC toolbox. The DAB color was extracted from each image, and the maximum and mean gray values were obtained. Each image’s optical density (OD) was obtained with log10(maximum gray value/mean gray value). The OD means and standard deviations were calculated and graphed. Student’s *t*-test was used to compare the expression at 24 h and 48 h.

CFA-injected footpads showed an inflammatory infiltrate 24 h post-injection, while those injected with SS had no infiltrate (Figure 4). In the tissues injected with CFA, the expression of CD4, CD8, and CD68 at 24 h was scarce and limited to the cells surrounding the oil drops, whereas, at 48 h, all of them were markedly increased (Figure 4). According to the analysis of the OD of the IHC images, the expression of these three markers was significantly higher at 48 h post-CFA injection.

**Figure 5:**
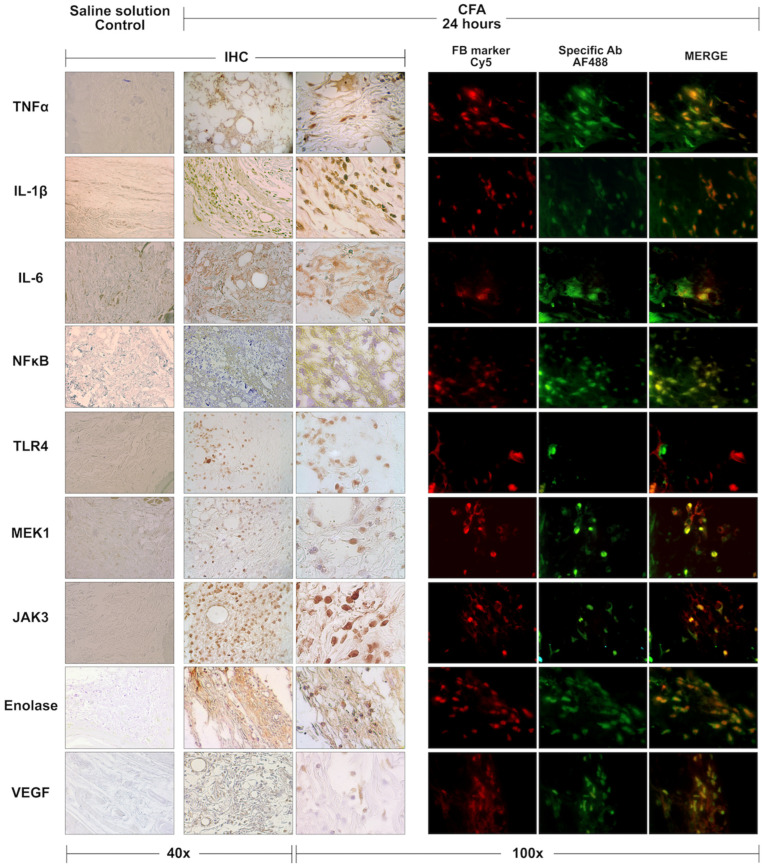
Immunodetection of Rheumatoid Arthritis related proteins in the rats’ footpads at 24 h post-Complete Freund’s adjuvant injection. TNF-α, IL-1β, IL-6, NFκB, TLR4, MEK1, JAK3, Eno, and VEGF proteins were identified in tissues using immunohistochemistry staining and photographed at 40× and 100× magnifications to observe tissue and cell positive detections. Double immunofluorescence was performed to co-localize these proteins (green) in the fibroblasts (ER-TR7; red).

Some relevant markers in the RA signaling pathways highlighted in the bioinformatic analysis were present in the CFA-injected footpads at 24 h post-injection. The positive immunodetections of TNFα, IL-1β, IL-6, NF-κB, TLR-4, MEK-1, Jak-3, Eno, and VEGF are shown in Figure 5. The staining patterns were different for each protein. Its expression was not exclusive to the inflammatory cells, showing clear patterns of expression in the skin, adipose tissue, and muscle. The double IIF stains confirmed that, except for the TLR4 marker, all those proteins were expressed in the fibroblast of CFA-injected footpads’ (Figure 5).

**Figure 6:**
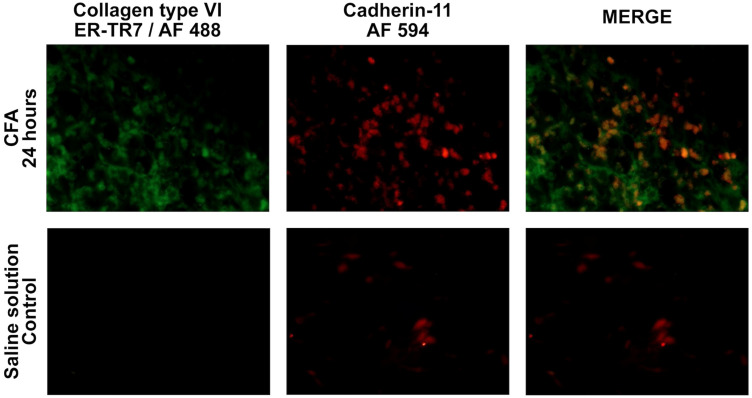
Immunodetection of Cadherin-11 and Collagen type VI in the rats’ footpads at 24 h post-Complete Freund’s adjuvant injection. Immunofluorescence was performed to co-localize Cadherin-11 (red) and Collagen type VI (fibroblast marker ER-TR7; green).

Moreover, the expression of CDH11 and ColVI, molecules associated with the pathogenic phenotype of FLS, were overexpressed in the cells from the CFA-injection site at 24 h (Figure 6). In the case of CDH11, an increased number of cells expressing it was noted compared to the SS-injected group. At the same time, the expression of ColVI only was confirmed in CFA-injected footpads.

## 4. Discussion

The present article describes the effect of CFA within the resident cells surrounding the injection in the subcutaneous footpad at the earliest time. Our results show that the CFA induces the activation of quiescent subcutaneous fibroblasts, which express a hypoxic metabolic profile and can produce key pathogenic inflammatory mediators for both AA and RA. This immunometabolic profile recreates the FLS phenotype [9,21,40] and suggests its critical role in the processes that explain AA onset. Although the involvement of FLS and its pathogenic phenotype in RA within the synovium has been widely studied, the potential participation and transformation of fibroblasts residing in distant tissues as precursors of these FLS remain undefined. Our findings confirm that in AA, from 24 h, the fibroblasts at the CFA-inoculation site expressed genes and proteins explicitly linked to RA. Moreover, we found that the footpad’s fibroblast overexpressed migration and invasion molecules related to RA-FLS.

Our results show that just 24 h after the injection, CFA dysregulated key signaling pathways linked to RA pathogenesis, including MAPK [41], Jak-STAT [42], HIF [43], PI3K-Akt [44], TLR [45], TNF [46], and NF-kB [47]. Likewise, our bioinformatic analysis revealed the most relevant genes in these pathways, including Mapk 1, 9, and 13, IL-1b, -2 and -6, Pik3r2, and NfκB. These findings are consistent with those described by Stolina M. et al. [48], which assessed the presence of biomarkers in 14 stages of the AA model, the earliest the day -5 concerning the onset of clinical arthritis. On the other hand, *Yu H.* et al. [49] reported that the most dramatic changes in gene expression were observed in the preclinical phase of the disease (day 7 post-injection). Exploring earlier times than previously reported, our study allows us to conclude that the processes that explain arthritis are established much earlier than was known.

The transcriptomic profiles at 24 and 48 h presented some differences worth noting. At 24 h, the transcriptional modifications were primarily associated with metabolic rather than inflammatory signaling pathways. Although at 24 h, the differential expression of some inflammatory cytokines such as Il1b was observed, the bioinformatic analysis indicated that, at this time, the associations with the high number of genes were of metabolic processes. In contrast, the transcriptome analyses at 48 h resulted in the most substantial identification of inflammatory processes and pathways. Indeed, the expression profile at 48 h was highly similar to what we previously observed in the joints, weeks after CFA injection, in other of our previous works carried out in this model [50].

Moreover, from 24 h, the neuroactive ligand-receptor interaction signaling pathway was deregulated by the CFA. Nine genes with an FDR of 1.9 × 10^−10^ were associated with this pathway at 24 h; notably, at 48 h, this pathway doubled the number of genes, and the FDR value decreased to 4 × 10^−24^. The neuroactive ligand-receptor interaction signaling pathway is directly related to neuro function. Neuroactive ligands influence neuronal function by binding to intracellular receptors, which can bind transcription factors and regulate gene expressions [51]. Recent studies have demonstrated that RA progression is closely related to abnormal function of the central nervous system. The nervous system (NS) can receive stimulation from immune cells. At the same time, the signals of the central NS act on immune cells through the peripheral NS to regulate the inflammatory response. In RA, cytokines such as IL-6, IL-1β, IL-17, and TNF can interact with joint nociceptors and activate and sensitize them. IL-1β may also be involved in RA-induced pain and hypersensitivity [52]. The above could suggest that the AA activation that begins at the inoculation site in the footpad is closely linked to neuronal processes from the early times of the disease.

At 24 h post-injection, our IHC analysis showed that CD4 and CD8 positive cells were virtually absent, and CD68 cells were scarce and limited to the periphery of the oil drops in CFA-injected footpads. Since our main objective was to describe the activation profile of fibroblasts at the CFA-injected footpads, we analyzed the expression of RA-metabolic and RA-inflammation markers at 24 h to isolate them as much as possible from the immune cells. The markers selection for IHC analysis resulted mainly from the microarray analyses. We choose markers highlighted at 24 and 48 h, such as IL-1β, MEK-1 (MAPK activator), JAK3, NF-κB, and Eno3. Other markers were selected because they are critical in RA-associated pathways, such as TNFα for the TNF signaling pathway, TLR4 for the TLR signaling pathway, and VEGF for the VEGF signaling pathway.

Compared with saline solution-injected footpads, at 24 h in the CFA-injected, the TNFα, IL-1β, IL-6, NF-κB, MEK-1, JAK-3, Eno, and VEGF proteins were overexpressed. The IHC showed that different types of cells expressed these markers; therefore, we performed the colocalization staining using a fibroblast marker. Our IIF confirmed that the footpad’s resident fibroblast expressed the markers of interest, suggesting their phenotype transformation resembling descriptions of FLS.

In RA, FLS exhibits a significant phenotype transformation; studying the metabolic and regulatory changes that drive this transformation is a promising field for understanding its etiopathogenesis. The FLS activation by hypoxia, platelet-derived growth factor, TNF, and other inflammatory mediators increases glucose metabolism and transforms the FLS from quiescent to aggressive and metabolically active cells. Glucose metabolism is increased in activated FLS, and glycolytic inhibition reduces FLS aggressive phenotype in vitro and decreases bone and cartilage damage in several murine models of arthritis [53,54,55]. In our study, at 24 h and 48 h, the CFA-injected footpads had dysregulated the HIF-1, TNF, and glycolysis signaling pathways. Essential genes, including Hk1, Gck, Aldoc, and Eno3, were increased by CFA injection; Eno3 expression was also confirmed in the fibroblast of footpads by IIF.

FLS also produces MMP-3, VEGF, and IL-6, which contribute to the worsening of arthritic conditions through the recruitment and activation of inflammatory cells and angiogenesis [12]. Moreover, angiogenesis is also linked to hypoxia and oxidative stress in RA [43,56]. Here, we also demonstrated that angiogenesis’ transcriptome was altered in the CFA-injected footpads; moreover, we confirmed that resident fibroblast expressed VEGF and IL-6.

Increasing evidence has demonstrated the role of mitochondrial alterations in RA mainly due to the interplay between metabolism and innate immunity and the modulation of the inflammatory response of FLS. Mitochondrial dysfunction derived from several danger signals could activate tricarboxylic acid (TCA) disruption, creating a vicious cycle of oxidative-mitochondrial stress [57]. Our microarray analyses showed that several mitochondrial metabolism genes, including Ak3, Idh3a, Idh3B, Idh3g, Asl, and Acyl, were deregulated by the CFA, suggesting the alteration of TCA.

In RA-FLS, secreted Frizzle-related protein-1 (SFPR1) regulates pyroptosis through WNT/beta-catenin and Notch signaling pathways [20]. Consistent with this description, the WNT pathway was dysregulated in CFA-injected footpads. In addition, other pathways widely recognized as altered in FLS, including Jak-STAT [58], PI3K-Akt, MAPK, and Toll-like receptors [21], were dysregulated by the CFA in our study. Furthermore, critical markers of these pathways, including MEK-1, JAK-3, and TLR4, are overexpressed by rat footpad fibroblasts after adjuvant injection.

Regarding the role of specific proinflammatory cytokines in the FLS pathogenic phenotype, it is known that IL-1β induces the proliferation of FLS through the mediation of the NF-κB signaling pathway [59]. The dysregulation of proinflammatory pathways was also found in our transcriptomic analyses. We found that Il1b and Nfkb genes were overexpressed in the footpads at 24 h and 48 h post-CFA injection. Moreover, we confirmed that at 24 h, the resident fibroblasts expressed both proteins.

Although arthritis is the hallmark of RA, it is a systemic disease, and the inflammation involves other organs and structures; therefore, the specific origin of the illness is a matter of great interest. Indeed, the possibility that the initial inflammatory process could start away from the joints is a valid research hypothesis; and the mucosal structures such as the gut [60,61], the lungs [62,63,64,65], or the oral cavity [66,67,68] are candidate triggering sites. If that is the case, a prerequisite is the migration of this initial priming to the susceptible joints.

The ability of MSC, fibrocytes, and fibroblasts to migrate and induce the pathogenic phenotype of FLS has gained evidence. In experimental arthritis, joint inflammation is preceded by infiltration of MSC, which contributes to synovial membrane hyperplasia. On the other hand, fibroblast migration in human disease has only recently been reported [22]. Fibrocytes were the first cells with fibroblastic properties described to migrate from the circulation into the joint [23]. Even activated fibrocytes are considered FLS precursors [24]. Some authors also supposed that FLS could migrate from one joint to another and potentially spread the disease; however, this idea remains unproven [69]. Recently, the presence of circulating fibroblast-like cells in the blood of patients with RA weeks before the disease flare-up was demonstrated. These cells are identified as PRe-Inflammatory MEsenchymal (PRIME) cells and share some markers with FLS, such as CDH11 [70].

CDH11 is mainly expressed in MSC and is essential for tissue migration and organization during embryogenesis [71]. In joints, CDH11 is primarily expressed in FLS and cooperates with inflammatory factors to promote the migration, invasion, and degradation of joint tissue in RA [72,73,74]. CDH11 was previously linked to ColVI in human MSC differentiation towards the adipogenic lineage [75] and adipose tissue fibroblasts [76]. Human MSCs lacking CDH11 had decreased the expression of ColVI and increased the expression of fibronectin through the TGFβ1 pathway [75]. Furthermore, in adipose tissue fibroblasts, CDH11 deficiency reduced their production of ColIII and ColVI, resulting in substantially less adipose tissue fibrosis in obesity [76]. ColVI is a structural and signaling protein that may act as an early sensor of the injury/repair response and regulate fibrogenesis by modulating cell-cell interactions and stimulating the proliferation of MSC. Moreover, ColVI regulates pericellular matrix properties, chondrocyte swelling, and mechanotransduction in articular cartilage [77,78]. In the RA synovium, ColVI is extensively deposited in the interstitial connective tissue and along the synovial membrane lining [79].

Remarkably, this interaction between CDH11 and ColVI (the antigen recognized by the ER-TR7 antibody [37,38] was observed in our study at CFA injection sites from 24 h, suggesting that footpad cells acquired a phenotype previously described for FLS. Considering that CDH11 has been associated with the fibroblast’s ability to migrate and invade the joint, our finding supports the possibility that these footpad’s fibroblasts initiate the pathogenic mechanisms. Interestingly, although CDH11 expression was markedly higher in cells from CFA-injected footpads, it was also detected in cells from the control group. Conversely, ColVI expression was only detected in CFA-injected foot pads and not controls. This finding suggests that CFA induces a fibrotic phenotype. Furthermore, fibrosis is associated with ColVI overexpression in a model of lung sepsis in rats within the first 24 h of LPS administration [80]. The above also allows us to suppose that the mycobacterial components of CFA could induce the fibrotic phenotype in our study.

## 5. Conclusions

The earliest events in AA include the dysregulation of several key pathogenic signaling pathways in the footpad residing tissular cells, including fibroblasts. These pathways are linked to established pathogenic pathways that explain joint inflammation and destruction. Aside from an evident metabolic dysregulation, the CFA induces a severe challenge to the tissue environment, which drives a significant adaptation in the cells’ behavior, with a widespread activation of protective mechanisms, including those that result in inflammation, migration, and fibrosis.

The fact that this transformation occurs in tissular fibroblast and that endorses it to acquire a phenotype shared with FLS becomes a matter of interest because it opens the potential to study its dynamics. It also reinforces its potential role as a critical player, under the logic of the Danger model, as a potential driving engine for the abnormal immune response resulting in arthritis.
